# Novel hydrazone compounds with broad-spectrum antiplasmodial activity and synergistic interactions with antimalarial drugs

**DOI:** 10.1128/aac.01643-23

**Published:** 2024-04-19

**Authors:** Angélica M. Rosado-Quiñones, Emilee E. Colón-Lorenzo, Zarna Rajeshkumar Pala, Jürgen Bosch, Karl Kudyba, Heather Kudyba, Susan E. Leed, Norma Roncal, Abel Baerga-Ortiz, Abiel Roche-Lima, Yamil Gerena, David A. Fidock, Alison Roth, Joel Vega-Rodríguez, Adelfa E. Serrano

**Affiliations:** 1 Department of Microbiology and Medical Zoology, University of Puerto Rico School of Medicine, San Juan, Puerto Rico; 2 Laboratory of Malaria and Vector Research, National Institute of Allergy and Infectious Diseases, National Institutes of Health, Rockville, Maryland, USA; 3 Center for Global Health and Diseases, Case Western Reserve University, Cleveland, Ohio, USA; 4 InterRayBio, LLC, Cleveland, Ohio, USA; 5 Department of Drug Discovery, Experimental Therapeutics Branch, Walter Reed Army Institute of Research, Silver Spring, Maryland, USA; 6 Department of Biochemistry, University of Puerto Rico School of Medicine, San Juan, Puerto Rico; 7 RCMI Program, Medical Science Campus, University of Puerto Rico, San Juan, Puerto Rico; 8 Department of Pharmacology and Toxicology, University of Puerto Rico School of Medicine, San Juan, Puerto Rico; 9 Department of Microbiology and Immunology, Columbia University, New York, New York, USA; 10 Division of Infectious Diseases, Department of Medicine, Center for Malaria Therapeutics and Antimicrobial Resistance, Columbia University Medical Center, New York, New York, USA; The Children's Hospital of Philadelphia, Philadelphia, Pennsylvania, USA

**Keywords:** malaria, *Plasmodium falciparum*, *Plasmodium berghei*, multistage activity, drug combinations

## Abstract

The development of novel antiplasmodial compounds with broad-spectrum activity against different stages of *Plasmodium* parasites is crucial to prevent malaria disease and parasite transmission. This study evaluated the antiplasmodial activity of seven novel hydrazone compounds (referred to as CB compounds: CB-27, CB-41, CB-50, CB-53, CB-58, CB-59, and CB-61) against multiple stages of *Plasmodium* parasites. All CB compounds inhibited blood stage proliferation of drug-resistant or sensitive strains of *Plasmodium falciparum* in the low micromolar to nanomolar range. Interestingly, CB-41 exhibited prophylactic activity against hypnozoites and liver schizonts in *Plasmodium cynomolgi*, a primate model for *Plasmodium vivax*. Four CB compounds (CB-27, CB-41, CB-53, and CB-61) inhibited *P. falciparum* oocyst formation in mosquitoes*,* and five CB compounds (CB-27, CB-41, CB-53, CB-58, and CB-61) hindered the *in vitro* development of *Plasmodium berghei* ookinetes. The CB compounds did not inhibit the activation of *P. berghei* female and male gametocytes *in vitro*. Isobologram assays demonstrated synergistic interactions between CB-61 and the FDA-approved antimalarial drugs, clindamycin and halofantrine. Testing of six CB compounds showed no inhibition of *Plasmodium* glutathione S-transferase as a putative target and no cytotoxicity in HepG2 liver cells. CB compounds are promising candidates for further development as antimalarial drugs against multidrug-resistant parasites, which could also prevent malaria transmission.

## INTRODUCTION

Malaria continues to be one of the leading causes of death from infectious diseases in endemic areas worldwide, with 247 million cases and 619,000 deaths in 2021 (1). The emergence of multidrug-resistant *Plasmodium falciparum* has significantly worsened the global malaria burden, with increasing incidence and mortality rates (2). Antimalarial monotherapies like chloroquine (CQ) (3–7) and artemisinin (ART) (8–11) have lost their efficacy, prompting the development of novel compounds for combination therapy. The development of new antimalarial drugs is urgently needed to overcome *P. falciparum* resistance and the incidence of malaria.

Malaria is a parasitic protozoan infection caused by *Plasmodium* parasites and transmitted by *Anopheles* mosquitoes. Sporozoites, the infectious stage transmitted by mosquitoes, invade the liver and develop into schizonts, releasing merozoites into the bloodstream. Merozoites invade red blood cells where asexual replication takes place. Some parasites differentiate into gametocytes, which are ingested by mosquitoes when they bite an infected person. The gametocytes form gametes in the mosquito, where they fertilize to form a zygote and then a motile ookinete that invades the midgut epithelium to form an oocyst. The oocyst undergoes sporogony to form sporozoites that invade the mosquito salivary glands. Sporozoites are then transmitted to a new host by a mosquito bite. Historically, antimalarial drugs targeted parasite stages within erythrocytes, neglecting transmission stages in mosquitoes (12). However, liver and mosquito stages represent crucial bottlenecks in the parasite’s life cycle, offering the potential for novel transmission-blocking drugs that target multiple stages to interrupt malaria transmission (13).

In searching for new drug targets, we previously identified *Plasmodium* glutathione S-transferase (GST) as an essential erythrocytic protein (14). GST is an important component of cellular detoxification by glutathione conjugation to xenobiotic compounds to increase their solubility and facilitate their excretion from the parasite (15, 16). Using structure-based screening and biological assays, our laboratory previously identified CB-27 as a novel *Plasmodium* GST inhibitor predicted to act at the species-specific H-site (14). Using CB-27 as a query in a shape similarity screening, we also discovered six antiplasmodial compounds (CB-41, CB-50, CB-53, CB-58, CB-59, and CB-61) (14). These CB compounds demonstrated potent antiplasmodial activities *in vitro* against *Plasmodium berghei* intraerythrocytic stages without causing erythrocyte lysis (14).

In this study, we report the antiplasmodial activities of seven CB compounds (CB-27, CB-41, CB-50, CB-53, CB-58, CB-59, and CB-61) against multiple *Plasmodium* stages and species. The compounds were active against *P. falciparum* drug-resistant and drug-sensitive asexual blood stage strains, multiple *Plasmodium* mosquito stages, and liver stages in the *Plasmodium cynomolgi* model. Notably, CB-61 showed synergistic interactions with the FDA-approved antimalarial drugs clindamycin (CLIND) and halofantrine (HALO). Unexpectedly, the six CB compounds did not inhibit their predicted *Plasmodium* GST target. The CB compounds did not demonstrate hepatocytotoxicity. The CB compounds represent a promising advance in developing novel antimalarial candidates as drugs to treat and prevent onward transmission of infection.

## RESULTS

### *Plasmodium* glutathione S-transferase target inhibition by CB compounds

**Fig 1:**
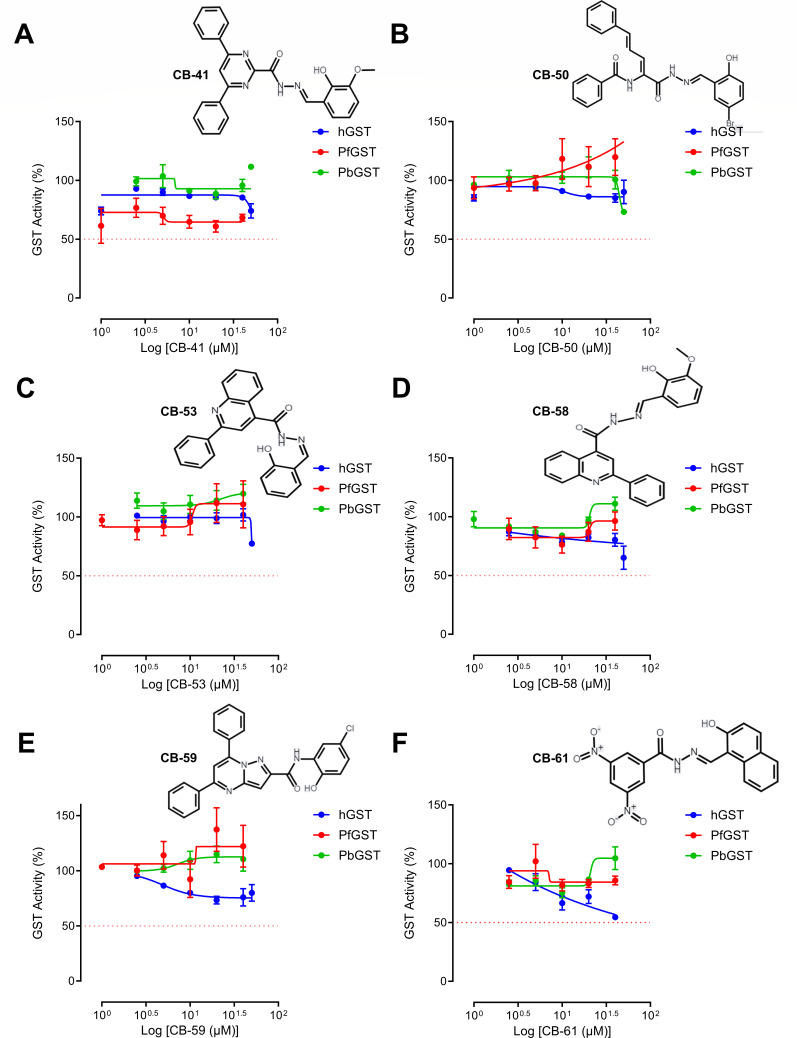
*Plasmodium* GST enzymatic inhibition assays with six candidate GST inhibitors. (**A–F**) Inhibition was assessed using recombinant *P. falciparum* GST (red), *P. berghei* GST (green), and human GST (blue) (0.35 mg/mL) in 6-point (1–50 µM) dose–response curves. The red dashed lines indicate the 50% inhibition cutoff. To calculate the IC_50_ scores, a nonlinear regression function was used for sigmoidal dose–response (variable slope). Positive controls (CB-27 and hemin) are included in Figure S1. Data are presented as mean ± SEM from four independent experiments performed in triplicate.

The inhibition of *Plasmodium* GST by CB compounds (CB-41, CB-50, CB-53, CB-58, CB-59, and CB-61) was assessed by enzymatic inhibition assays using recombinant GST (Fig. 1). Results show that the six CB compounds did not inhibit either *P. falciparum*, *P. berghei*, or human GST (Fig. 1). *Plasmodium* GST inhibition was confirmed by CB-27, which inhibited the activity of recombinant GST from *P. falciparum* (half-maximal inhibitory concentration [IC_50_] = 22.9 µM) and *P. berghei* (IC_50_ = 21.1 µM) but had no activity against human GST. Hemin, used as a positive control, inhibited human (IC_50_ = <1 µM), *P. falciparum* (IC_50_ = 1.6 µM), and *P. berghei* (IC_50_ = 16.1 µM) recombinant GST (Fig. S1). These findings suggest that the six CB compounds tested do not target *Plasmodium* GST.

### Antiplasmodial activity of CB compounds: *P. falciparum* blood stages and *P. cynomolgi* liver stages

To determine whether CB compounds inhibit the blood stages of *P. falciparum*, their activities were examined against drug-sensitive (D6) or drug-resistant (TM91c235, W2, and C2B) *P. falciparum* strains. Three resistant strains and one sensitive strain were selected, including TM91c235 (resistant to cycloguanil, pyrimethamine [PYR], mefloquine, sulfadoxine, and quinine, but sensitive to WR99210 and CQ), the Cambodia strain W2 (CQ resistant), the Thai laboratory strain C2B (resistant to atovaquone [ATQ] and sensitive to ART), and the Sierra Leone D6 sensitive strain (17). All CB compounds showed antiplasmodial activity against blood stages of resistant and sensitive strains (Table 1). Interestingly, CB-27 and CB-61 were remarkably active with IC_50_ values in the nanomolar range. Overall, these data show that all CB compounds inhibit the blood stages of *P. falciparum-*sensitive and resistant strains.

**TABLE 1:** Antiplasmodial activities of CB compounds in the blood stages of *P. falciparum*-sensitive (D6 and TM91c235) and resistant strains (TM91c235, W2, and C2B)^*a*^^,^^*b*^

Compound	IC_50_ (µM) (±SD) for strain:			
	D6	TM91c235	W2	C2B
CB-27	0.28 (±0.19)	0.38 (±0.31)	0.31 (±0.18)	0.40 (±0.24)
CB-41	2.26 (±1.15)	2.26 (±1.10)	2.71 (±1.87)	2.73 (±1.19)
CB-50	1.14 (±0.82)	1.84 (±0.68)	1.15 (±0.59)	1.89 (±1.29)
CB-53	>20	13.89 (±3.56)	>20	>20
CB-58	8.44 (±2.48)	10.62 (±3.05)	5.63 (±2.56)	8.83 (±3.33)
CB-59	1.73 (±1.47)	2.96 (±3.84)	0.69 (±1.08)	2.91 (±1.35)
CB-61	0.20 (±0.05)	0.26 (±0.03)	0.22 (±0.03)	0.30 (±0.12)

^*a*^ Dose–response results are summarized in IC_50_ values and standard deviations (SD). These experiments were carried out in two independent experiments performed in triplicate.

^*b*^ *P. falciparum* strains and sensitivity profiles: D6 = susceptible to CQ, PYR, cycloguanil, and WR99210 (dihydrofolate reductase inhibitor); TM91c235 = susceptible to WR99210 but resistant to cycloguanil, PYR, mefloquine, sulfadoxine, and quinine; W2 = Indochina clone, showing resistance to CQ resistance; C2B = ATQ resistant.

*P. cynomolgi,* a primate malaria model closely related to *Plasmodium vivax*, shares the ability to form dormant liver stages called hypnozoites. These hypnozoites are responsible for recurrent *P. vivax* malaria episodes in humans. We used *in vitro P. cynomolgi* liver-stage cultures to investigate the activity of CB compounds (18). Interestingly, CB-41 showed some activity against *P. cynomolgi* hepatic schizonts (IC_50_: 8.7 µM) and hypnozoites (IC_50_: 8.4 µM) stages in the prophylactic drug treatment mode (Table 2). Conversely, CB-41 did not show antiplasmodial activity in the radical cure mode. The remaining six CB compounds did not inhibit the growth of *P. cynomolgi* liver-stage parasites in either the prophylactic or the radical cure mode (Table 2). No hepatocyte toxicity was observed, as evidenced by the preserved viability of primary hepatocytes at concentrations >10 µM. The findings suggest that CB-41 could be used to prevent the development and relapse of malaria from the liver stages.

**TABLE 2:** Prophylactic antiplasmodial activity of CB-41 in hypnozoites and hepatic schizonts in the *P. cynomolgi* model^*a*^

Compound	Toxicity (µM)	Hypnozoite IC_50_ (µM)	Schizont IC_50_ (µM)	Total parasite IC_50_ (µM)
CB-27	>10	>10	>10	>10
CB-41	>10	8.7	8.4	8.6
CB-50	>10	>10	>10	>10
CB-53	>10	>10	>10	>10
CB-58	>10	>10	>10	>10
CB-59	>10	>10	>10	>10
CB-61	>10	>10	>10	>10

^*a*^ These experiments were performed in one independent experiment, triplicate for the prophylactic drug treatment mode, and duplicate for the radical cure mode.

### Antiplasmodial activity of CB compounds: *Plasmodium* gametes, ookinetes, and oocysts stages

**Fig 2:**
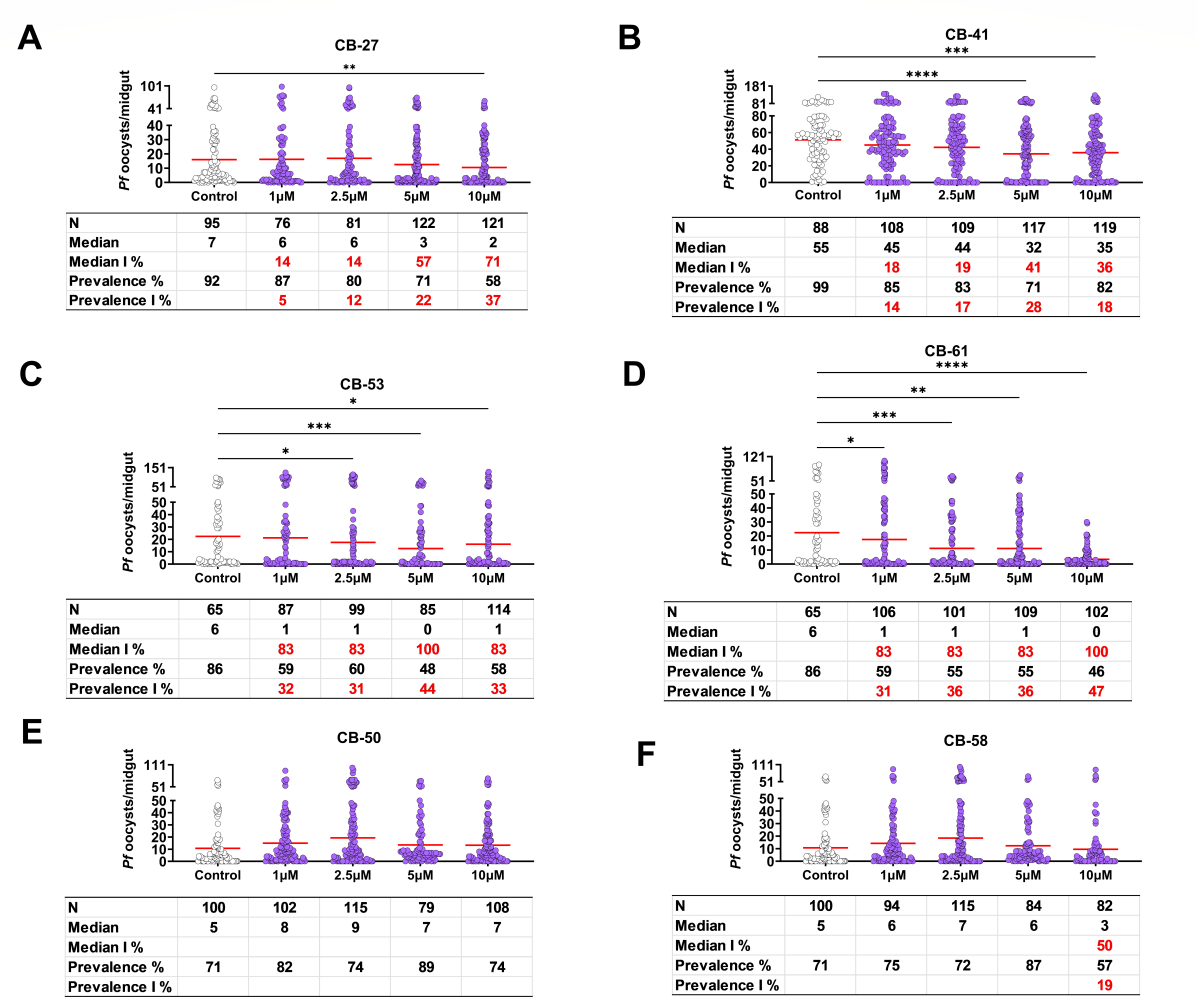
Antiplasmodial activity of CB compounds on *P. falciparum* oocyst formation. *Anopheles stephensi* mosquitoes were fed with *P. falciparum* infectious blood supplemented with increasing concentrations (1 to 10 µM) of the CB compounds or DMSO control using SMFA. Horizontal red lines represent the median. Data were pooled from two representative experiments. Additional experiments are shown in Table S2. Statistical analysis was performed using the Kruskal-Wallis test with the Dunn post-test. *P < 0.021, **P < 0.0021, ***P < 0.00021, and ****P < 0.000021. Median I%, median inhibition percentage; N, number of mosquitoes dissected; median I % or prevalence I %: 100 × ([number of positives in control – number of positives in experimental]/[number of oocysts in control]). (A) CB-27, (B) CB-41, (C) CB-53, (D) CB-61, (E) CB-50, (F) CB-58.

To assess the activity of the CB compounds against *P. falciparum* oocyst formation, mosquitoes were fed with infected blood containing mature gametocytes and supplemented with CB compounds in a standard membrane-feeding assay (SMFA). Mosquito midguts were dissected 8 days after feeding to determine the number of oocysts. CB-41 and CB-61 inhibited midgut oocyst formation in a dose-dependent manner, while CB-27 and CB-53 did not (Fig. 2A through D; Tables S 2–S3). Although CB-41 statistically inhibited oocyst formation compared to the control, its median and prevalence inhibition percentages were below 50% (Fig. 2B; Table S2-S3), indicating weak activity against oocysts. The results show that CB compounds CB-50 and CB-58 did not inhibit oocyst formation (Fig. 2E and F;Table S2–S3).

**Fig 3:**
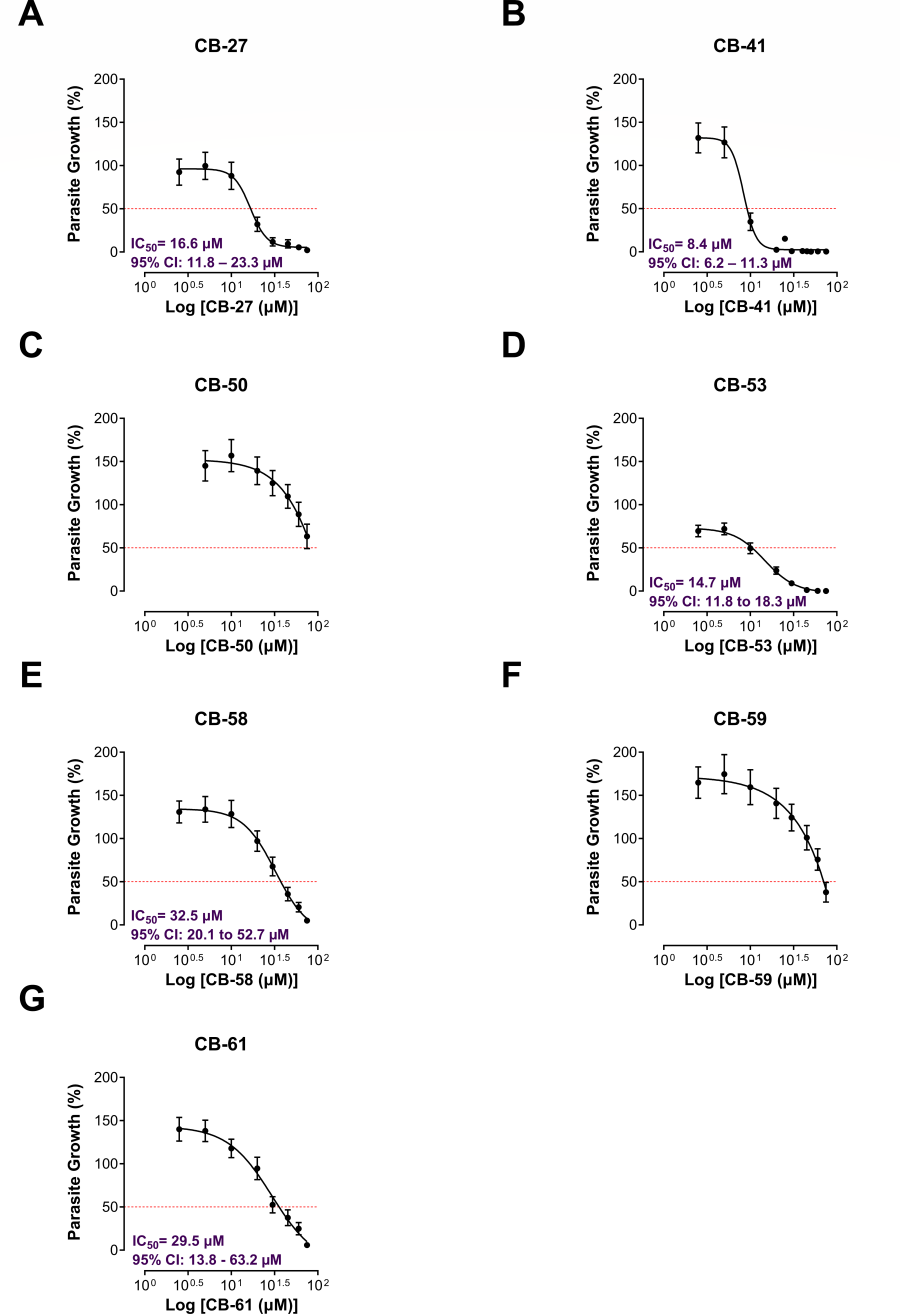
Antiplasmodial activity of CB compounds on *P. berghei* ookinete development. Ookinete maturation was evaluated by quantifying the luciferase activity expressed by Ookluc parasites, which harbor the luciferase gene driven by the ookinete-specific *ctrp* gene promoter. The red dashed lines indicate the 50% inhibition cutoff. To calculate the IC_50_ scores, a nonlinear regression function was used for sigmoidal dose–response (variable slope). Data are presented as mean ± SEM and represent three independent experiments each performed in triplicate. (A) CB-27, (B) CB-41, (C) CB-50, (D), CB-53, (E) CB-58, (F) CB-59,(G) CB-61.

CB compound activity against *P. berghei* ookinete development was measured *in vitro* using the luminescence signal from Ookluc parasites. As Ookluc expresses luciferase under the ookinete-specific *ctrp* promoter, luminescence directly reflects the ookinete number. Dose–response curves with 6-point concentrations were generated for each CB compound. Results demonstrate that five CB antiplasmodial compounds (CB-27, CB-41, CB-53, CB-58, and CB-61) inhibit ookinete development (Fig. 3A through G). CB-27 exhibited the lowest IC_50_ (9.2 µM) of the CB compounds, followed by CB-53 (14.7 µM), CB-41 (16.6 µM), CB-61 (29.5 µM), and CB-58 (32.5 µM). CB-50 and CB-59 showed no activity in ookinete formation (Fig. 3C and F). These results demonstrated that five CB compounds inhibit *P. berghei* ookinete *in vitro* development, suggesting that CB compounds act in the *Plasmodium* mosquito stages after fertilization.

**Fig 4:**
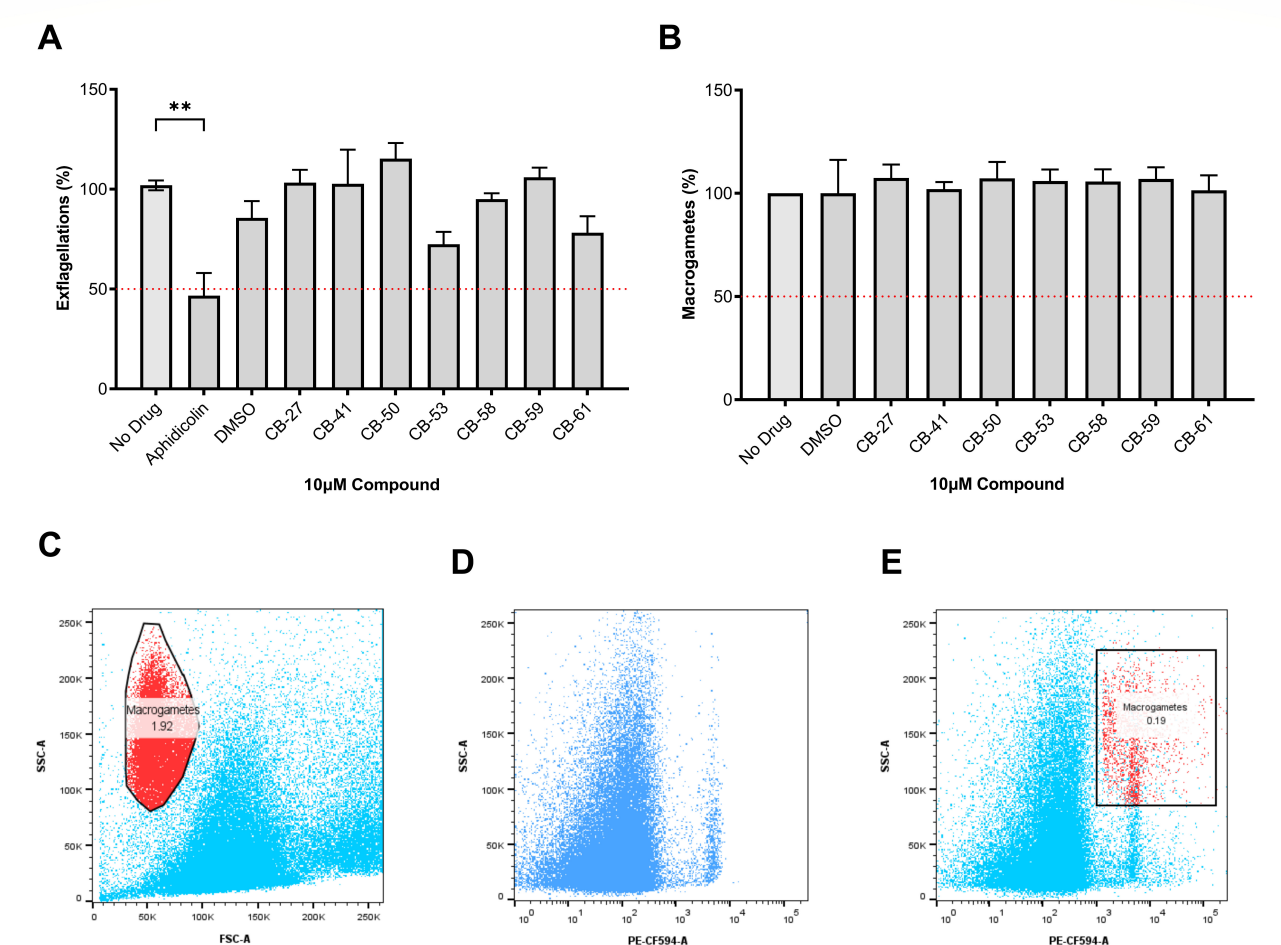
Antiplasmodial activity of CB compounds on *P. berghei* male and female gametocyte activation. (**A**) Exflagellation assays. Aphidicolin (125 nM), a DNA polymerase inhibitor, was a positive control to block exflagellations (19). (**B**) Macrogamete flow cytometry assays were carried out to determine *in vitro* inhibition of CB compounds on female gametocyte activation. Female gametes were detected by staining with antibodies against the surface protein Pbs21. The red dashed lines indicate the 50% inhibition cutoff. Data are presented as mean ± SEM and represent three independent experiments performed in triplicate. Statistical analysis was conducted using one-way ANOVA with Dunnett’s multiple comparison tests. The significance level was **P < 0.0021. (**C–E**) Gating strategy for flow cytometry analysis of *P. berghei* macrogametes (red – %) labeled with anti-Pbs21 antibody and Alexa Fluor 594 secondary antibody from a pool of red blood cells and immune cells (blue). (**C**) Side (SCC-A) and forward scatter area (FSC-A) dot plot of *P. berghei* macrogametes in culture. (**D**) SSC-A vs PE-CF594 dot plot of *P. berghei* macrogametes in culture with no antibodies (no fluorescent control). (**E**) SSC-A vs PE-CF594 dot plot of *P. berghei* macrogametes in culture with both antibodies.

The activity of CB compounds was evaluated for inhibition of *P. berghei* male and female gamete activation. Male gamete activation was assessed by inducing exflagellation in the presence of each CB compound at 10 µM for 15 minutes and counting exflagellations by microscopy (Fig. 4A). The results revealed that none of the CB compounds affected *P. berghei* exflagellations, while aphidicolin, a DNA polymerase inhibitor used as a positive control, showed a significant reduction in the number of exflagellation events (<50%) (Fig. 4A). To determine their activity against female gamete activation, infected blood was incubated with 10 µM of each CB compound for 2 hours and then analyzed by flow cytometry. The macrogamete subpopulation was identified by staining with antibodies against the surface marker Pbs21, which is only produced in activated female gametes and zygotes (Fig. 4C through E). The CB compounds did not inhibit the activation of *P. berghei* female gametes (Fig. 4B). Collectively, these data demonstrated that CB compounds do not affect male and female gamete activation. These results indicate that CB-27, CB-41, CB-53, and CB-61 inhibit the development of oocysts in the mosquito midgut, representing potential transmission-blocking antiplasmodial drugs.

### *In vitro* cytotoxicity of CB compounds in HepG2 cells

A counter-screen with uninfected HepG2 mammalian cells was performed to assess the potential cytotoxicity and selectivity index (SI) of CB compounds (CB-27, CB-41, CB-50, CB-53, CB-58, CB-59, and CB-61) in human liver cells. No hepatotoxicity of the CB compounds was observed at the highest concentration tested (20.2 µM) in HepG2 hepatoma cells when compared to the IC_50_ of the positive control WR288510-3 (1.3 µM) (UPSOT 7259167B2) (Table 3). The SI (20, 21), a decisive metric signifying the antimalarial selectivity of the CB compounds in relation to the positive control WR288510-3, exceeds the 100-fold threshold (Table 3). These findings suggest that the CB compounds did not induce host hepatotoxicity.

**TABLE 3:** Cytotoxicity and SI of CB compounds in the mammalian HepG2 hepatoma cell line^*b*^

Compound	HepG2 cytotox (µM)	SI (relative to WR288510-3^*a*^)
CB-27	>20	>100-fold
CB-41	>20	>100-fold
CB-50	>20	>100-fold
CB-53	>20	>100-fold
CB-58	>20	>100-fold
CB-59	>20	>100-fold
CB-61	>20	>100-fold
WR288510-3^*a*^	1.28	1-fold

^*a*^ Positive control.

^*b*^ Data represent two independent experiments.

### Antiplasmodial synergistic interactions: CB-61 and FDA-approved antimalarial drugs

**Fig 5:**
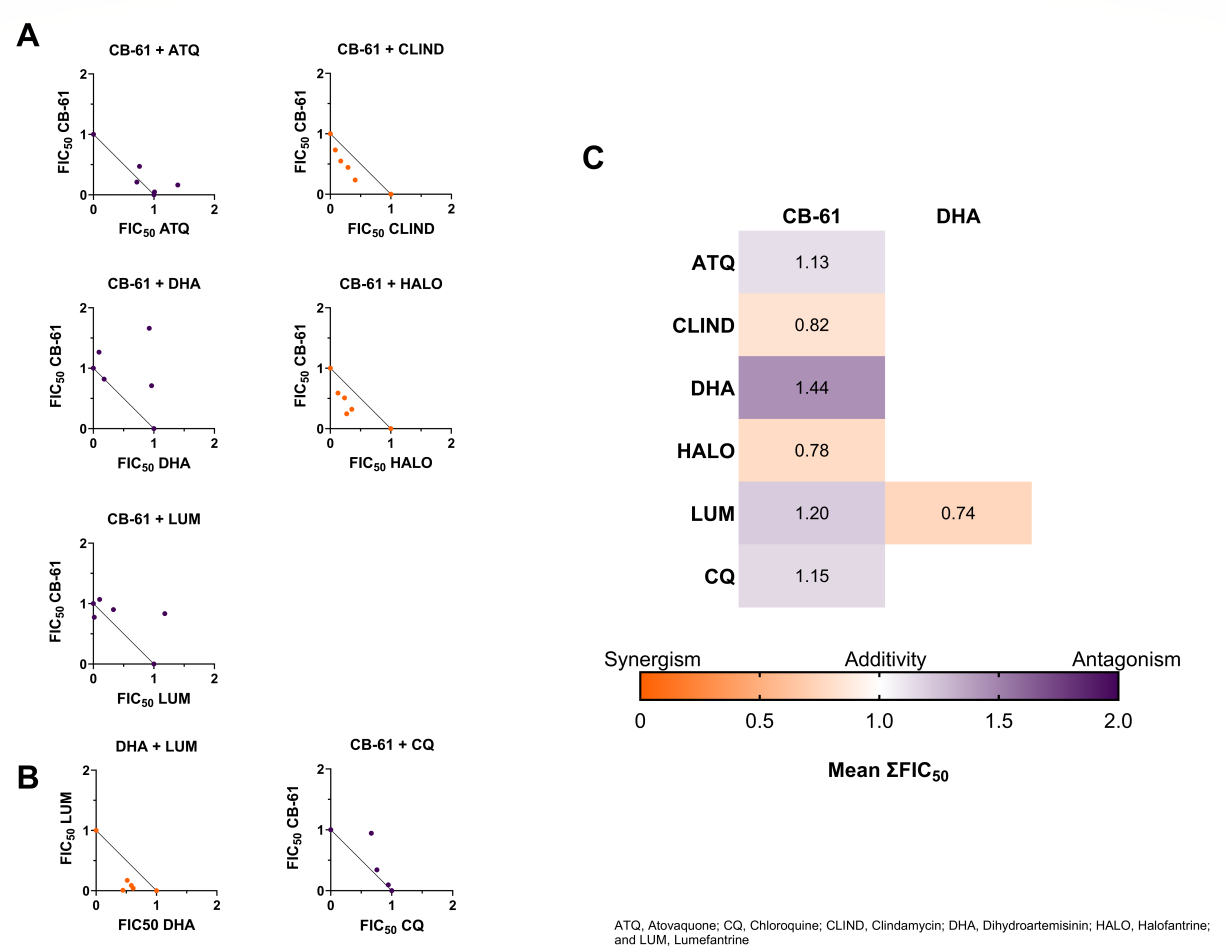
Isobolograms of drug combinations of CB-61 with FDA-approved antimalarial drugs. (**A**) Isobolograms of CB-61 with ATQ, CLIND, DHA, HALO, and LUM. (**B**) Isobolograms of positive and negative controls. The *P. berghei* blood-stage parasites were exposed to drug combinations in fixed ratios of their IC_50_ scores (5:0, 4:1, 3:2, 2:3, 1:4, 0:5). FIC_50_ values were plotted for each drug combination and the fixed ratio. The results were compared against a full black line representing the perfect additive trend (1,1). The classifiers used were synergy FIC_50_ < 1 (orange); additivity FIC_50_ = 1 (white); and antagonism FIC_50_ > 1 (purple). (**C**) Heatmaps of the means of the sum of FIC_50_ (∑FIC_50_) of the five experimental and the two control combinations. LUM-DHA is a proven synergistic combination (positive control), and CB-61-CQ displays an antagonistic interaction (negative control). Data are presented as means of the sums of the FIC_50_ scores and represent three independent experiments performed in triplicate. ATQ, atovaquone; CQ, chloroquine; CLIND, clindamycin; DHA, dihydroartemisinin; HALO, halofantrine; and LUM, lumefantrine.

Synergistic combinations between CB-61 and 85 FDA-approved antimalarial drugs were predicted using the Machine Learning Synergy Predictor tool (MLSyPred) (22). Five potential synergistic combinations were predicted (Fig. S2), and isobolograms were used to validate antiplasmodial synergistic predictions in *P. berghei* blood stages. The synergistic antimalarial drugs predicted were ATQ, CLIND, dihydroartemisinin (DHA), HALO, and lumefantrine (LUM). Isobologram analyses show the interactions of the drug combinations in terms of synergism, additive, and antagonism. In the isobologram, points located significantly below the line connecting the half-maximal fractional inhibitory concentration (FIC_50_) of 1 from both compounds (diagonal black line) indicate a synergistic interaction, while points close to the line suggest an additive interaction. Conversely, points well above the line signify an antagonistic interaction. Results showed that CB-61 displayed antiplasmodial synergistic interactions with CLIND and HALO (Fig. 5A). In contrast, antagonistic interactions were detected between CB-61 and ATQ, DHA, and LUM (Fig. 5A). As predicted, the positive control combination (LUM-DHA) exhibited synergy, whereas the negative control combination (CB-61 and CQ) showed antagonism (Fig. 5B). The results are shown as the mean of the sums of the FIC_50_ values per combination (mean ΣFIC_50_) and are represented in heatmaps (Fig. 5C). The precision of the MLSyPred tool was improved to 57%, potentially representing a valuable tool for predicting the synergistic combinations of antimalarial drugs (Table 4). The findings suggest that CB-61 has the potential to be a synergistic partner with select antimalarial drugs.

**TABLE 4:** Machine learning predictions obtained by MLSyPred, an open-source tool to predict synergistic antimalarial drug combinations and their subsequent validation using *P. berghei* isobolograms

Compound	Compound	Prediction	Validation
CB-61	ATQ	Synergy	Antagonism
CB-61	CLIND	Synergy	Synergy
CB-61	DHA	Synergy	Antagonism
CB-61	HALO	Synergy	Synergy
CB-61	LUM	Synergy	Antagonism
CB-61	CQ	No synergy	Antagonism
LUM^*a*^	DHA^*a*^	Synergy	Synergy

^*a*^ Positive control; a proven synergistic interaction (23).

## DISCUSSION

The development of novel antimalarial drugs with activity against the multiple stages of the *Plasmodium* parasite is crucial to combating multidrug-resistant parasites and preventing malaria transmission. New antimalarial drugs that target different *Plasmodium* species stages can improve treatment efficacy, reduce the risk of resistance development, and potentially eliminate the parasite from both humans and mosquitoes. Our previous work identified CB-27, a specific inhibitor of *P. berghei* GST, active in *P. falciparum*, and six additional CB compounds that inhibit *P. berghei* erythrocytic stages (14). Herein, we report the broad-spectrum activity of the seven novel antiplasmodial CB compounds (CB-27, CB-41, CB-50, CB-53, CB-58, CB-59, and CB-61) on blood, liver, and mosquito stages of *Plasmodium* species parasites. The potential for synergistic interactions in combinatorial therapy was demonstrated. Our results reveal that the CB compounds are promising new candidates for the development of multistage antimalarial drugs.

The CB compounds were selected *in silico* to target the *Plasmodium* GST. The identified CB-27 compound was predicted and demonstrated to inhibit *Plasmodium* GST (14), an attractive target because it is directly involved in detoxifying reactive oxygen species (15, 16). Our previous work has shown that *Plasmodium* gamma-glutamylcysteine synthetase and glutathione reductase are critical for mosquito-stage survival, and the *Plasmodium* cytosolic GST is an essential protein in blood-stage parasites, representing a good target for the development of novel compounds (14, 16, 20–22).

In a previous study, our laboratory identified six CB compounds by shape similarity screening using CB-27, a *Plasmodium* GST inhibitor, as a query. The identified CB-27 compound was predicted and demonstrated to inhibit *Plasmodium* GST (14). Our results, however, show no inhibition of *Plasmodium* GST by the six CB compounds analyzed. Nonetheless, all CB compounds exhibited multistage and multispecies *Plasmodium* inhibition, suggesting they target other proteins or pathways essential for *Plasmodium* species survival. The discrepancy between predicted and validated inhibition of *Plasmodium* GST by the six CB compounds likely arises from the Rapid Overlay of Chemical Structures (ROCS) similarity score limitations. Although ROCS scores assess similarity to known inhibitors based on binding affinity, toxicity, and other properties, they have been shown to be inaccurate for some compounds (18, 24–26). Studies on HIV-1 proteases and phosphoinositide 3 kinase delta identified few molecules exhibiting inhibitory activity and low IC_50_ values despite high ROCS scores, suggesting that shape similarity alone may not be sufficient for accurate prediction (25, 26). Specific inhibitor tools utilizing large data sets and focusing on shared structural features and binding affinities could offer a more practical approach to identifying potent inhibitors (18, 24–27).

Despite the limitations of ROCS similarity screening, we discovered CB-27 as a putative *Plasmodium* GST inhibitor (14). CB-27 differs in structure and mode of action from known GST inhibitors such as hemin and S-hexylglutathione. Hemin, an iron-containing porphyrin derived from heme, binds the GST G-site, inhibiting glutathione conjugation due to its planar structure (28–33). Meanwhile, S-hexylglutathione, a hexyl chloride glutathione conjugate, binds both G and H sites with its non-planar structure, further enhancing inhibition (28, 29, 33, 34). The *Plasmodium* GST inhibitor CB-27 is a hydrazone compound predicted to bind the *Plasmodium* species-specific H-site without affecting the evolutionary preserved G-site (14, 35). In this study, we further validated the inhibition of *Plasmodium* GST by CB-27 using recombinant proteins, specifically by measuring the enzymatic activity of recombinant *Plasmodium* GST. Further studies are needed to determine the target pathway of the remaining CB compounds and optimize their properties for preclinical development.

This study investigated the activity of the CB compounds against the blood stages of *P. falciparum-*resistant and sensitive strains. Our results revealed that all seven CB compounds inhibited the growth of *P. falciparum* blood stages in all four strains tested. The most potent inhibitors were CB-27 and CB-61, which inhibited parasite growth at nanomolar concentrations in all strains. Further research on CB compounds will be needed to determine whether or not the parasite can develop resistance to these drugs. Our findings suggest that CB compounds, especially CB-27 and CB-61, represent promising antiplasmodial candidates that can clear asexual blood-stage parasitemia.

Inhibition of both liver schizonts and hypnozoites is an important attribute of CB-41. *P. vivax* and *Plasmodium ovale* are the only human malaria parasites with hypnozoite stages, which can persist in hepatocytes and cause relapse of infection days, weeks, months, and years after the end of drug treatment (36, 37). Both primaquine and tafenoquine are 8-aminoquinoline drugs approved by the FDA against anti-hepatic schizonts and hypnozoites, which prevent *P. vivax* relapse (38–40). Unfortunately, primaquine is underused worldwide as it can cause hemolytic anemia in individuals with low glucose-6-phosphate dehydrogenase activity. Safer treatments are urgently required (41). Identifying novel compounds active against hypnozoites and liver-stage schizonts in *Plasmodium* relapsing models is crucial to malaria eradication. Most recently, other novel *P. vivax* hypnozoite and hepatic schizont inhibitors have reported IC_50_ values under 10 µM, but none were tested in multiple *Plasmodium* species and stages (42, 43). Structural modification of CB-41 can help improve its activity. CB-41 is our first compound to initially show activity against both *P. cynomolgi* liver schizonts and hypnozoites *in vitro*.

Primaquine is the only licensed antimalarial drug recommended by WHO that kills late-stage gametocytes and prevents *P. vivax* relapse (39). In this study, we evaluated the activity of CB compounds in blood gametocytes and transmission stages in the mosquito host. CB-27, CB-41, CB-53, and CB-61 inhibited oocyst formation in mosquitoes, thus possessing the criteria to block transmission stages. We also observed that CB-27, CB-41, CB-53, CB-58, and CB-61 blocked *P. berghei* ookinete development in a dose-dependent manner. The CB compounds did not affect *P. berghei* micro- and macro-gamete formation when incubated with gametocytes at the moment of the assay. Our data show that CB compounds are active against mosquito stages after fertilization during ookinete and oocyst formation.

Determining the pharmacokinetics and toxicity of a compound is a crucial step in the early stages of drug development because this information can be used to prioritize promising drug candidates and optimize the development of lead compounds (44). Previously, we showed that the CB compounds do not induce hemolysis of erythrocytes (14). The CB compounds demonstrated no toxicity in mammalian cells, as evidenced by their lack of cytotoxicity against the HepG2 hepatoma cell line at concentrations up to 20.2 µM. Similarly, no toxicity was observed in primary hepatocytes in the *P. cynomolgi* liver-stage assays. Previous ADMET (Absorption, Distribution, Metabolism, Excretion, Toxicity) predictions revealed that CB compounds had hepatoxicity similar to CQ (14). Further studies are needed to evaluate the potential long-term effects of CB compounds on host cells prior to preclinical testing.

Combination therapy has been central to preventing resistance against the few antimalarial drugs available. In this study, we explored potential partner drugs for CB-61 combination therapies. In this study, 85 antimalarial drugs were selected and tested for synergistic interactions with CB-61. Drug combinations can display three primary types of interactions: synergism, additivity, and antagonism. Synergism occurs when the combined effect of two or more drugs exceeds the sum of their actions, leading to enhanced therapeutic outcomes and potentially reduced drug dosages (45–48). Additivity signifies that the combined effect of drugs is similar to the sum of their individual actions, neither enhancing nor diminishing each other’s actions. This neutral interaction is represented by the additive (1,1) line in isobologram graphs (46). In contrast, antagonism arises when the combined effect of two or more drugs is less than the sum of their actions, causing a weakened therapeutic response (46). Fixed-ratio isobologram studies revealed synergy between CB-61 and two antimalarial drugs: CLIND and HALO. CLIND is a repurposed drug that binds to the 50s bacterial ribosomal subunit, disrupting protein synthesis (49). Meanwhile, HALO hinders the polymerization of heme molecules, and the parasite is poisoned with its waste products (50). These structurally diverse drugs have different mechanisms of action that can work in unison with CB-61, thus creating synergy. The synergistic drug interactions of CB-61 make it an attractive candidate for combination therapies that can clear the pathogenic asexual blood stages and prevent transmission.

We report here a series of CB compounds (CB-27, CB-41, CB-50, CB-53, CB 58, CB-59, and CB-61) that are lead candidates for further investigation as broad-spectrum antimalarial drugs. Our results revealed greater efficacy against drug-resistant and sensitive *Plasmodium* blood stages. Remarkably, the CB compounds also exhibit antiplasmodial activity against mosquito and liver stages, emerging as promising therapeutic leads for malaria.

## MATERIALS AND METHODS

### Parasites, cell lines, antibodies, and reagents

*P. berghei* GFP-Lucama1, expressing the green fluorescent protein and firefly luciferase genes under the control of the ama-1 schizont-specific promoter, was provided by the Leiden University Malaria Research Group and was utilized for isobologram experiments (51). Similarly, *P. berghei* Ookluc, expressing luciferase under the *P. berghei* circumsporozoite protein and thrombospondin-related adhesive protein (CTRP) promoter, was obtained from Daniel Bargieri and Richard Eastman from the NIH and was employed in experiments involving macrogametes, microgametes, and ookinetes (52). Maintenance of mosquitoes and *P. falciparum* was performed as previously described (53). Laboratory strains of *P. falciparum* (D6, TM91-C235, W2, and C2B) were maintained and used for *in vitro* Malaria SYBR Green I-Based Fluorescence (MSF) assays. The *Anopheles dirus* mosquitoes harboring *P. cynomolgi bastianellii* (B strain) sporozoites were used to infect the primary primate hepatocytes, which were obtained cryopreserved from BioIVT Inc. (Baltimore, MD, USA) and maintained in hepatocyte culture medium (HCM) (InVitroGro CP medium). Recombinant GST proteins from *P. falciparum* (PfGST), *P. berghei* (PbGST), and human (hGST) were obtained from InterRayBio, LLC (Cleaveland, OH, USA) and were utilized for GST inhibition assays. The CB compounds (CB-27, CB-41, CB-50, CB-53, CB-58, CB-59, and CB-61) were obtained from the ChemBridge Hit2lead library (San Diego, CA, USA). The anti-Pbs21 mouse monoclonal antibody, prepared in Joel Vega-Rodríguez laboratory at the NIH, and anti-mouse goat IgG conjugated Alexa Fluor 594, from Invitrogen, were used in immunofluorescence assays. Human hepatocarcinoma cells (HepG2) were cultured in complete Minimal Essential Medium (MEM) (Gibco-Invitrogen, #11090-099) supplemented with 0.19% sodium bicarbonate (Gibco-BRL), 10% heat-inactivated fetal bovine serum (Gibco-Invitrogen), 2 mM L-glutamine (Gibco-Invitrogen), 0.1 mM MEM nonessential amino acids (Gibco-Invitrogen), 0.009 mg/mL insulin, and 1.76 mg/mL bovine serum albumin. All reagents, unless otherwise stated, were purchased through Sigma (St. Louis, MO, USA).

### GST plasmid constructs

The open reading frames encoding PfGST, PbGST, and hGST were gene-synthesized and harmonized from their respective genomic DNA using sequence-specific sense oligonucleotides (Table S1). The sequences, produced as a geneblock by Integrated DNA Technologies Inc., were cloned into the *Escherichia coli pRSF-1b* expression vector with a KanR cassette. All recombinant proteins were codon-optimized for bacterial expression using JCat (54). The antisense oligonucleotide encoded a His10 tag for purification (HisTrap excel Ni Sepharose, Cytiva), a Tobacco Etch Virus cleavage site, and an Avitag with one lysine for biotinylation or streptavidin purification/surface plasmon resonance chip binding. The PfGST, PbGST, and hGST constructs consisted of 247, 241, and 258 amino acids with molecular weights of ~29.3, ~28.5, and ~30.1 kDa, respectively.

### GST expression and purification

Recombinant GST proteins were produced from frozen *E. coli* BL21 (DE3) cultures in Terrific Broth with kanamycin for 16 hours. Induction was triggered with isopropyl β-D-1-thiogalactopyranoside at OD600 3.0, followed by centrifugation (1,000 × *g* for 15 minutes at 25°C) and pellet freezing at −20°C. Pellets were resuspended in BugBuster, protein inhibitor cocktail, and benzonase nuclease, followed by 20 minutes of incubation and removal of insoluble cell debris by centrifugation (35 minutes at 12,000 × *g* at 4°C). GST proteins were purified by affinity chromatography using a HisTrap Excel Ni Sepharose column and an ÄKTA Go system (Cytiva) at a 1-mg/mL flow rate. The column was washed with distilled water and then sequentially with equilibration buffer (1 M phosphate buffer, 1 M NaCl, pH 7.4), protein lysate, wash buffer (25 mM Tris, 500 mM NaCl, 20 mM imidazole, 1 mM DTT), and elution buffer (500 mM imidazole, 20 mM sodium phosphate monobasic, 0.5 M NaCl, 1 mM DTT). Finally, the column was washed and prepared with distilled water and 20% ethanol. Western blot analysis confirmed the presence of the three GST proteins (1:1,000 PentaHis HRP Antibody, LI-COR WesternSure PREMIUM chemiluminescence). Pooled fractions containing GST proteins were concentrated using a 5-kDa cutoff polyethersulfone tube (1,000 × *g*, 3 cycles, 90 min/cycle) and were adjusted to pH 7.4.

### GST inhibition assay

Enzymatic activities of recombinant GSTs were determined spectrophotometrically using the chromogenic substrate 1-chloro-2,4-dinitrobenzene (CDNB). Recombinant GST (0.35 mg/mL) was mixed with 1 mM CDNB in 100 mM HEPES. Stock solutions (10 mM) of the CB compounds and hemin were prepared in 100% dimethyl sulfoxide (DMSO) and then diluted at 0.5% DMSO (14). Each inhibitor has six concentrations ranging from 1 to 50 µM. The reaction was initiated with 1 mM glutathione (GSH) after 15 minutes. Absorbance change at 340 nm was measured for 1 hour. The slope was converted to micromole per minute using Beer’s equation and the extinction coefficient for the product S-(2,4-dinitrophenyl)glutathione (ε340 nm = 9.6 mM^–1^ cm^–1^) (14). The assay was validated using human placenta GST (#G8642) as a positive control.

### *P. falciparum* SYBR Green drug sensitivity assessment

MSF assays were performed *in vitro* with four *P. falciparum* strains: D6 (drug sensitive), TM91-C235 (multidrug resistant), W2 (CQ resistant), and C2B (multidrug resistant against ATQ). Parasite strains were kept continuously in long-term cultures as described (22). Pre-dosed 384-well plates containing 12-fold serial dilutions of CB compounds (0.0098–20 µM) were prepared using the Tecan Freedom Evo liquid handling system (Tecan USA, Inc., Durham, NC, USA) and were stored at 4°C. A CQ control plate (2,000 ng/mL) was included in each run. Based on modifications of previous methods (17, 55), late-ring or early-trophozoite stages were cultured in pre-dosed plates with 0.3% parasitemia and 2% hematocrit. Cultures were incubated for 72 hours at 37°C in a controlled atmosphere of 5% CO_2_, 5% O_2_, and 90% N_2_. Lysis buffer, consisting of 20 mM Tris HCl, 5 mM EDTA, 1.6% Triton X, 0.016% saponin, and SYBR Green I dye at a 20X concentration (Invitrogen #S-7567), was added to the plates for a final 10X SYBR Green concentration. Plates containing cell culture media and lysis buffer were incubated in the dark at 25°C for 24 hours, and the relative fluorescence units (RFU) were measured using the Tecan Genios Plus (Tecan USA, Inc.).

### *P. cynomolgi* liver-stage inhibition assay

Primary primate hepatocytes were seeded in 384-well plates and used within 2–4 days (56). *P. cynomolgi* B strain parasites were inoculated and developed into hypnozoites and schizonts. CB compounds were tested in 8-point, threefold serial dilutions (100 µM to 5 nM) for both prophylactic (4 days starting with sporozoite addition) and radical cure (4 days starting day 4 post-inoculation) modalities (56–60). The Operetta CLS imaging system and Harmony Software 4.9 (Perkin Elmer, Waltham, MA, USA) analyzed the plates, and the percentage of inhibition (PI) was calculated in Python using equation (1), where parasitic quantities (hypnozoite and schizont counts) were normalized to the negative control (57). Each plate included negative (DMSO) and positive (KDU691, maduramicin, tafenoquine) controls (58). IC_50_ values were determined using an 8-point, threefold dilution format and a Python-adapted grid algorithm for fitting PI to a four-parameter logistic function (61).

Equation (1) Percent inhibition


$$PI\;=\;100\;*\left(1-\left({\frac{xy}{Mean}}_{neg}\right)\right)$$

### *P. falciparum* oocyst inhibition assay

As previously described*, P. falciparum* infections were performed using SMFA with NF54 gametocyte cultures diluted to 0.3% stage V gametocytemia (62). Infected blood (O+ human 45% hematocrit in serum) was offered to mosquitoes for 30 minutes, and engorged mosquitoes were maintained on a 10% corn syrup solution supplemented by four concentrations of CB compounds (1, 2.5, 5, and 10 µM) at 37°C (63), with DMSO serving as a control. Eight days post-infection, mosquito midguts were dissected and stained with 0.2% mercurochrome for 30 minutes, and oocysts were quantified under a light microscope (63).

### *P. berghei* ookinete luciferase inhibition assay

Mice were pretreated with phenylhydrazine [6 mg/mL, intraperitoneal injection (IP)] 3 days before intravenous injection (IV) infection with *P. berghei* Ookluc parasites (19, 62). Once parasitemia exceeded 15%, the mice received PYR (1 mg/mL, IP) (19, 62). Twenty-four hours after PYR treatment, infected mice were assessed for at least 10 exflagellations before blood was collected by heart puncture (19, 62). Infected blood (1:20 dilution) was incubated with 8-point serial dilutions (2.5–75 µM) of CB compounds for 24 hours at 19°C with gentle shaking (62, 64). Relative luminescence units (RLU) of mature ookinetes were measured using the Promega Nano-Glo Luciferase Assay System Kit and a SpectraMax M3 Microplate Reader (Molecular Devices, San Jose, CA, USA) after 3 minutes. Giemsa blood smears confirmed ookinetes.

### *P. berghei* gametocyte activation assays

Exflagellation assays were performed to assess the effect of CB compounds on *P. berghei* male gametocyte activation (19). Tail blood from PYR-administered mice was mixed with DMSO, aphidicolin (125 nM), or CB compounds (10 µM) in complete ookinete medium and heparin (30 µg/mL) (19). After a 15-minute incubation, the sample was diluted in complete ookinete medium with 10 µM inhibitor and adjusted to a final blood concentration of 1:20. Exflagellations were quantified by counting all events in the middle squares of a hemocytometer. The effect of CB compounds on macrogamete formation was analyzed by flow cytometry using the anti-Pbs21 antibody (65). Following PYR administration, infected blood for cardiac puncture was cultured with CB compounds (10 µM) for 2 hours to stimulate gametocyte activation (66). Aliquots were then incubated with anti-Pbs21 antibody (1:500) for 1 hour, followed by anti-mouse IgG conjugated Alexa Fluor 594 (1:1,000) for another hour (65). Samples were centrifuged (3,000 × *g* for 3 minutes), pellets were resuspended in phosphate-buffered saline (PBS) and analyzed by flow cytometry (FACSCelesta, FACS Diva Software, BD Biosciences, San Jose, CA, USA) with 1 million events counted per experiment (66).

### *In vitro* toxicity assessment in HepG2 cells

HepG2 cell viability was assessed using the Cell Proliferation kit with trypan blue exclusion. Cells were seeded at 2.5 × 10^4 per well in 96-well plates, incubated at 37°C in a humidified atmosphere of 5% CO_2_, and treated with 11 duplicate 1.6-fold serial dilutions of CB compounds (0.15–10 μg/mL) for 48 hours using a Biomek 4000 automated station. WR 288510-3 (IC_50_ = 305.4 ng/mL) was used as a positive control. After incubation, a 1.5-mg/mL Cell Proliferation kit solution diluted in complete MEM medium was added to each well, followed by 1-hour dark incubation at 25°C. After aspiration of media and inhibitors, the plates were dried in a hood for 15 minutes, and acidified isopropyl alcohol was added to dissolve formazan dye crystals. The plates were then rotated for 15–30 minutes, and absorbance was determined using a Perkin Elmer Ensight plate reader.

### Synergy predictor

The MLSyPred tool (https://github.com/rcmi-igpd/MLSyPred) (67) predicted synergistic combinations between CB-61 and 85 FDA-approved antimalarial drugs. The tool analyzed the SMILES (Simplified Molecular Input Line Entry System) chemical formulas of the compounds and predicted synergy for each of the three models of *P. falciparum*.

### *P. berghei* blood-stage isobolograms

Antimalarial activity and IC_50_ values of CB-61 and five antimalarial drugs were assessed using the previously described *in vitro* drug luminescence assay (14, 68). Blood-stage isobolograms were performed to confirm synergistic combinations between CB-61 and the five drugs, following established protocols (69). Thirty-two-fold IC_50_ concentrations were used to obtain six concentration ranges for each drug combination, and then they were serially diluted five times (67). IC_50_ scores for each drug alone were determined, and FIC_50_ values (FIC_50_ = IC_50_ of each drug alone/IC_50_ of each drug in combination) were calculated and plotted (70, 71). The Promega Luciferase Assay System Kit was used to measure mature schizonts’ RLUs, and Giemsa blood smears confirmed the presence of schizonts (14).

### Statistical analysis

Data were analyzed using GraphPad Prism Software 10.1.1. Dose–response curves were plotted, and IC_50_ values were calculated using a nonlinear regression function (14). Oocyst assays were analyzed using Kruskal-Wallis multiple comparisons with the Dunn post-test, and the chi-square test analyzed the oocyst prevalence (63). Gametocyte activation assays were analyzed with a one-way analysis of variance (ANOVA) using Dunnett’s multiple comparison tests (19). Differences were considered significant at *P* < 0.05.

## SUPPLEMENTAL MATERIAL

Supplemental figures aac.01643-23-s0001.pdf Figure S1 and S2.

Supplemental data aac.01643-23-s0002.xlsx Tables S1 to S3.

ASM does not own the copyrights to Supplemental Material that may be linked to, or accessed through, an article. The authors have granted ASM a non-exclusive, world-wide license to publish the Supplemental Material files. Please contact the corresponding author directly for reuse.
