# Supplementary material for: Novel hydrazone compounds with broad-spectrum antiplasmodial activity and synergistic interactions with antimalarial drugs
Source: Antimicrob Agents Chemother. 2024 Apr 19;68(6):e01643-23. doi: 10.1128/aac.01643-23 (PMC11620517; doi:10.1128/aac.01643-23)
Supplement: Supplemental figures — Figure S1 and S2. [file aac.01643-23-s0001.pdf]

Supplementary Figures: The authors declare that all relevant data supporting the findings of this study are available in the paper and provided as Supplementary Data 1-3 (Excel) and figures (Word).

Figure S1. *Plasmodium* GST inhibition assays with two known *Plasmodium* GST inhibitors: CB-27 and hemin. To confirm inhibition in recombinant *P. falciparum* GST (red), *P. berghei* GST (green), and human GST (blue) (0.35 mg/mL), 6-point (1 µM – 50 µM) dose-response curves were done for (A) CB-27 (*P. berghei* GST inhibitor) and (B) hemin (GST inhibitor) as positive controls. The red dashed line indicates the 50% inhibition cutoff. Data represent mean ± SD and show one independent experiment performed in triplicate. The IC_{50} values obtained for CB-27 and hemin were consistent with those reported in the literature (14, 31).

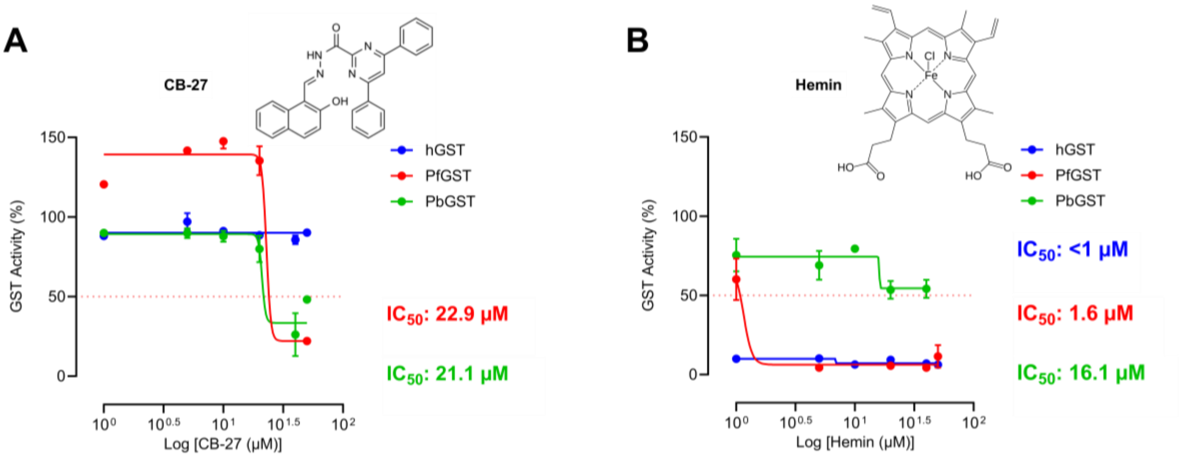

Figure S2. Heatmap of synergistic predictions of CB-61 in three *P. falciparum* strain (3D7, Dd2, and HB3) ML models from MLSyPred© (67). Eighty-five antimalarial drugs were chosen based on varying modes of action, drug structural composition, and drug type classification. Predictions were made using the SMILE chemical structures of CB-61 and antimalarial drugs from PubChem and ChemBridge. Drug combinations are identified based on the synergy prediction shared on three ML models of *P. falciparum* strains (dark pink), 2 strains (medium pink), 1 strain (light pink), and 0 strains (white). CB-61 has five predicted synergistic interactions.

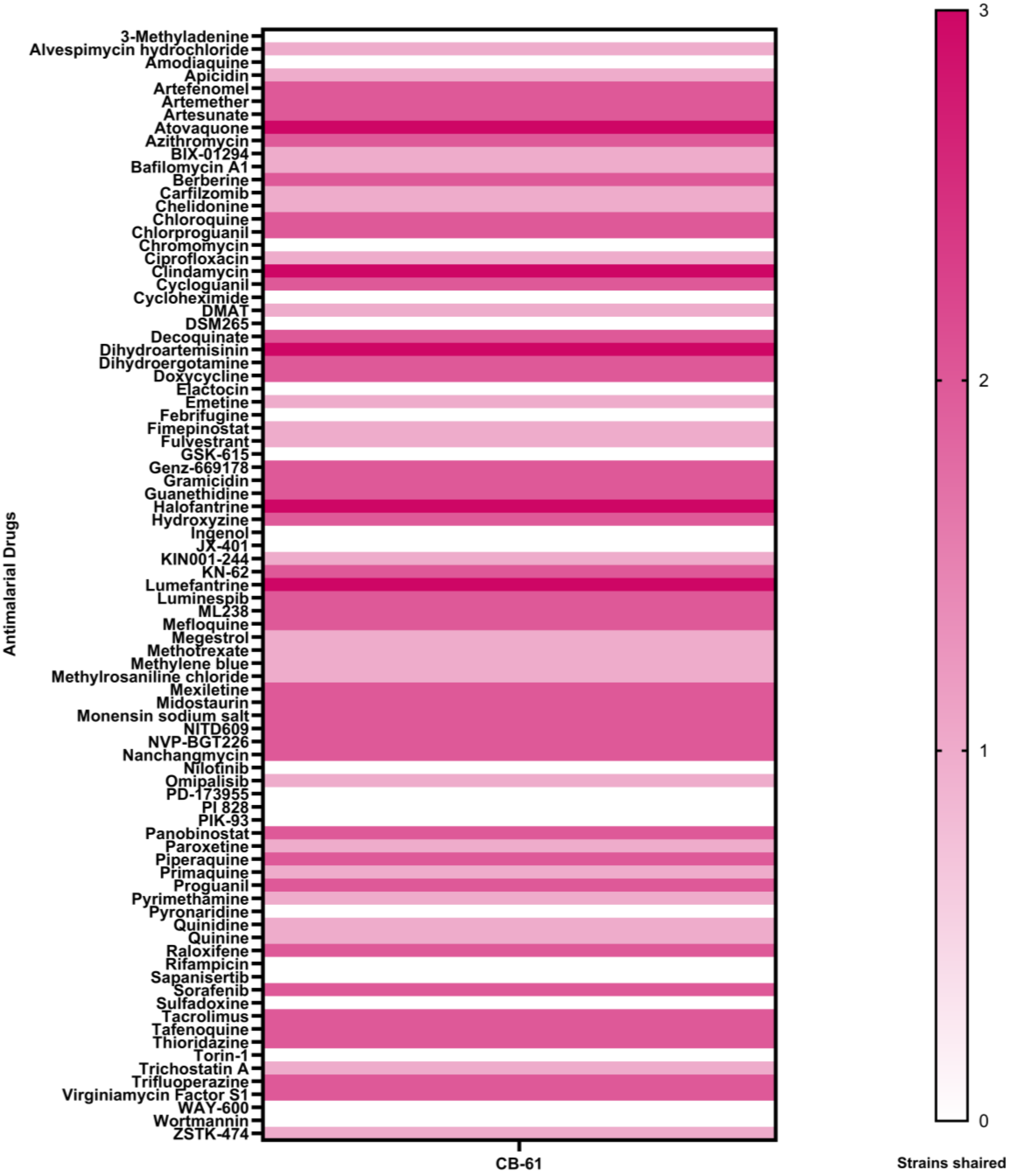
Antimalarial Drugs
3-Methyladenine
Alvespimycin hydrochloride
Amodiaquine
Apicidin
Artefenomel
Artemether
Artesunate
Atovaquone
Azithromycin
BIX-01294
Bafilomycin A1
Berberine
Carfilzomib
Chelidonine
Chloroquine
Chlorproguanil
Chromomycin
Ciprofloxacin
Clindamycin
Cycloguanil
Cycloheximide
DMAT
DSM265
Decoquinate
Dihydroartemisinin
Dihydroergotamine
Doxycycline
Elactocin
Emetine
Febrifugine
Fimepinostat
Fulvestrant
GSK-615
Genz-669178
Gramicidin
Guanethidine
Halofantrine
Hydroxyzine
Ingenol
JX-401
KIN001-244
KN-62
Lumefantrine
Luminespib
ML238
Mefloquine
Megestrol
Methotrexate
Methylene blue
Methylrosaniline chloride
Mexiletine
Midostaurin
Monensin sodium salt
NITD609
NVP-BGT226
Nanchangmycin
Nilotinib
Omipalisib
PD-173955
PI 828
PIK-93
Panobinostat
Paroxetine
Piperaquine
Primaquine
Proguanil
Pyrimethamine
Pyronaridine
Quinidine
Quinine
Raloxifene
Rifampicin
Sapanisertib
Sorafenib
Sulfadoxine
Tacrolimus
Tafenoquine
Thioridazine
Torin-1
Trichostatin A
Trifluoperazine
Virginiamycin Factor S1
WAY-600
Wortmannin
ZSTK-474
CB-61
3
2
1
0
Strains shaired
